# 1812. Comparative Activity of Oritavancin and Comparator Agents against Staphylococcus aureus and Streptococcus spp. Causing Skin and Skin Structure Infections in US Medical Centers between 2017–2019 and 2022

**DOI:** 10.1093/ofid/ofad500.1641

**Published:** 2023-11-27

**Authors:** Cecilia G Carvalhaes, Rodrigo E Mendes, Dee Shortridge, Mariana Castanheira

**Affiliations:** JMI Laboratories, North Liberty, IA; JMI Laboratories, North Liberty, IA; JMI Laboratories, North Liberty, IA; JMI Laboratories, North Liberty, IA

## Abstract

**Background:**

A new formulation of oritavancin (ORI) was developed and approved by the US FDA to be infused over 1 hour for the treatment of acute bacterial skin and skin structure infection (ABSSSI).

The activity of ORI and comparators against *Staphylococcus aureus* (SA), β-hemolytic streptococci (BHS), and Viridans group *Streptococcus* (VGS) causing SSSI in US medical centers in 2022 and 2017–2019 was compared.

**Figure:**
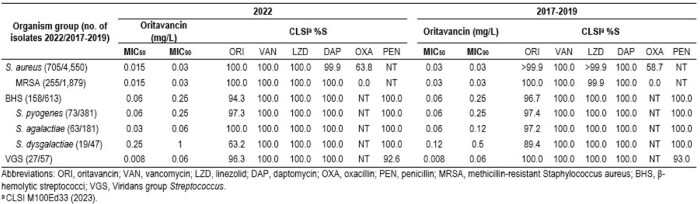

**Methods:**

6,110 SSSI pathogens, including 705/4,550 SA from 2022/2017–2019, 158/613 BHS, and 27/57 VHS isolates, respectively, were collected (1/patient) from 33 US medical centers. Isolates were identified by MALDI-TOF MS and susceptibility (S) tested by CLSI broth microdilution. CLSI clinical breakpoints (BP) were applied.

**Results:**

ORI activity against SA from 2022 (MIC_50/90_, 0.015/0.03 mg/L) was similar to 2017–2019 (MIC_50/90_, 0.03/0.03 mg/L; Table). The MRSA rate (36.2%) was slightly lower in 2022 than previous years (41.3%), but the ORI S rate remained 100% in 2022. Vancomycin (VAN), daptomycin (DAP), and linezolid (LZD) also remained active (≥ 99.9% S) against SA and MRSA from both periods. Equivalent activity was observed for ORI against the BHS group between 2022 (MIC_50/90_, 0.06/0.25 mg/L; 94.3%S) and 2017–2019 (MIC_50/90_, 0.06/0.25 mg/L; 96.7%S), but variation among species was noted. All *S. agalactiae* were inhibited by ORI at ≤ 0.25 mg/L in 2022 while 5 isolates showed ORI MIC values ≥ 0.5 mg/L in 2017–2019 (97.2%S). 2 and 10 *S. pyogenes* displayed ORI MICs ≥ 0.5 mg/L in 2022 (97.3%) and 2017–2019 (97.4%S), respectively. ORI MICs ≥ 0.5 mg/L were noted for 7/19 and 5/47 *S. dysgalactiae* in 2022 (63.2%S) and 2017–2019 (89.4%), respectively. Penicillin (PEN), VAN, DAP, and LZD inhibited all BHS isolates at their respective BP, regardless of the period or BHS species. All VGS were inhibited by ORI, VAN, DAP, LZD, and PEN in 2022 and 2017–2019, except for 1 *Streptococcus salivarius* group in 2022 (ORI MIC, 1 mg/L; 96.3%S).

**Conclusion:**

ORI data from 2022 showed similar activity to 2017–2019 for SA, including MRSA, BHS, and VGS isolates causing SSSI in US medical centers. VAN, DAP, and LZD also exhibited stable activity against these pathogens over time. *S. dysgalactiae* showed the largest changes in %S between periods but isolate numbers were low.

**Disclosures:**

**Cecilia G. Carvalhaes, MD, PhD**, AbbVie: Grant/Research Support|bioMerieux: Grant/Research Support|Cipla: Grant/Research Support|CorMedix: Grant/Research Support|Melinta: Grant/Research Support|Pfizer: Grant/Research Support **Rodrigo E. Mendes, PhD**, AbbVie: Grant/Research Support|Basilea: Grant/Research Support|Cipla: Grant/Research Support|Entasis: Grant/Research Support|GSK: Grant/Research Support|Paratek: Grant/Research Support|Pfizer: Grant/Research Support|Shionogi: Grant/Research Support **Dee Shortridge, PhD**, Melinta: Grant/Research Support|Shionogi: Grant/Research Support **Mariana Castanheira, PhD**, AbbVie: Grant/Research Support|Basilea: Grant/Research Support|bioMerieux: Grant/Research Support|Cipla: Grant/Research Support|CorMedix: Grant/Research Support|Entasis: Grant/Research Support|Melinta: Grant/Research Support|Paratek: Grant/Research Support|Pfizer: Grant/Research Support|Shionogi: Grant/Research Support

